# Metabolic Disorders in Menopause

**DOI:** 10.3390/metabo12100954

**Published:** 2022-10-08

**Authors:** Hye Gyeong Jeong, Hyuntae Park

**Affiliations:** 1 Department of Obstetrics and Gynecology, Korea University College of Medicine, 73 Inchon-ro, Seoul 02841, Korea; 2 Department of Obstetrics and Gynecology, Seoul National University College of Medicine, Seoul 03080, Korea

**Keywords:** menopause, metabolic disorder, metabolic syndrome, postmenopause

## Abstract

Menopause is an aging process and an important time equivalent to one-third of a woman’s lifetime. Menopause significantly increases the risk of cardiometabolic diseases, such as obesity, type 2 diabetes, cardiovascular diseases, non-alcoholic liver disease (NAFLD)/metabolic associated fatty liver disease (MFFLD), and metabolic syndrome (MetS). Women experience a variety of symptoms in the perimenopausal period, and these symptoms are distressing for most women. Many factors worsen a woman’s menopausal experience, and controlling these factors may be a strategy to improve postmenopausal women’s health. This review aimed to confirm the association between menopause and metabolic diseases (especially MetS), including pathophysiology, definition, prevalence, diagnosis, management, and prevention.

## 1. Introduction

Globally, the proportion of older adults is growing rapidly. According to United Nations reports, there were 54 million people aged 80 years and above worldwide in 1990, and the number was estimated to be around 143 million in 2019, a triple increase; it is predicted to reach 226 million by 2050. In other words, life expectancy was 72.6 years in 2019 but is expected to increase to 77.1 years by 2050 [1]. Life expectancy differs between the sexes; it has been shown to be longer in women than in men. In the United States in 1900, there were 102 men for every 100 women 65 and older. Currently, there are 70 men for every 100 women over the age of 65 years. By the age of 85 years, only 39 men are alive for every 100 women [2]. Differences in life expectancy between the sexes are a result of the high incidence of cardiovascular diseases in men due to differences in cholesterol-lipoprotein profiles and other cardiovascular factors caused by sex hormones [3]. 

Generally, menopause is clinically diagnosed when a woman has not menstruated for twelve months, which usually occurs around the age of 45–55 years [4,5]. Hormonal changes that begin at the menopausal transition and abrupt cessation of hormone production affect many biological systems. Signs and symptoms of menopause include metabolic, weight, cardiovascular, and musculoskeletal changes; genitourinary and skin atrophy; sexual dysfunction; and central-nervous-system-related disorders [6,7]. Although the physiological bases for these symptoms is complex and interrelated, the symptoms are not solely caused by estrogen deficiency. Among them, metabolic disorders are closely related, chronic, and progressive and greatly affect the health of postmenopausal women [8]. The increase in life expectancy, especially in women, increases the time a woman has to live following menopause while simultaneously increasing interest in its impact on various health problems during the postmenopausal period [9,10].

In this review, we investigated the metabolic disorders, especially metabolic syndrome (MetS), that could occur in postmenopausal women and confirmed their causes, diagnoses, and treatment methods to determine which approach is needed for healthy living in postmenopausal women.

## 2. Definition of Menopause and Metabolic Disorders

### 2.1. Menopause

Menopause is a result of aging, which essentially means that the ovaries are no longer ovulating or producing very few sex hormones [11,12]. Clinically, natural menopause is defined as a 1-year period of amenorrhea following the final menstrual period (FMP) without any pathological or physiological cause [13,14]. 

The median age of menopause was reportedly 51.3 years in the Massachusetts Women’s Health Study [15], and smoking may hasten menopause by 1.5 years, thus causing premature menopause. Factors that do not affect age at menopause include use of oral contraceptives and socioeconomic and marital status. The average age of menopause was reportedly 50.7 years in the Treloar study, 50.2 years in the Dutch study, 50.9 years in the Italian study, and 51.4 years in the SWAN study [16,17,18].

Abrupt cessation of menstruation-related hormone production affects bones (especially density) and several organs, including the heart and vessels; it even precipitates some cancers [19,20]. Estrogen is the primary sex hormone in women, and it affects development and functioning of the female reproductive system and organs, such as the breasts, vagina, and uterus [21]. Various symptoms related to menopause reportedly affect women’s lives. Among the known symptoms of menopause, central-nervous-system-related symptoms include vasomotor symptoms, sleep disturbance, depression and anxiety, cognitive changes, and migraines [22]. In addition, weight and metabolic changes, cardiovascular changes, genitourinary symptoms, sexual dysfunction, musculoskeletal symptoms, skin, mucous membrane, hair changes, and personal and social impacts are menopausal symptoms [8]. Body weight, metabolic, and cardiovascular changes are chronic and progressive in menopause, and management of chronic non-infectious diseases is important because it extends life expectancy [23].

The definition of premature menopause is failure of ovarian function before the age of 40 years prematurely, and it occurs in approximately 1% of women in the general population [24]. Premature menopause could be idiopathic for no reason or could be induced by specific causes. Some cases of premature menopause are associated with autoimmune or genetic diseases, infections, enzyme deficiencies, and MetS. Causes of induced premature menopause include chemotherapy and surgical procedure (i.e., bilateral oophorectomy). The importance of premature menopause is being emphasized as it is associated with an increased risk of MetS, cardiovascular disease (CVD), non-alcoholic liver disease (NAFLD)/metabolic associated fatty liver disease (MFFLD), stroke, osteoporosis, and premature death [24,25]. 

### 2.2. Change of Metabolism in Menopausal Women

Aging is associated with an increase in obesity [26], CVD, type 2 diabetes, hypertension, and non-inflammatory diseases, such as stroke [27]. The increased risk of non-infectious diseases is associated with an increase in central obesity and waist circumference (WC) and does not appear to be related to general obesity or an increase in body mass index (BMI) itself [27]. Particularly in women, changes in fat before and after menopause change central adiposity [28]. 

Menopause is associated with quantitative and morphological changes in adipose tissue (e.g., increased abdominal adiposity), as well as an altered lipid profile and onset of insulin resistance (IR) [11]. According to longitudinal studies, menopausal transition is characterized by a shift from a predominantly estrogenic state to an androgenic state due to increased levels of bioavailable testosterone [8,29]. Ovaries continue to produce large amounts of androgens for several years after menopause, and increased gonadotropin levels induce ovarian androgen secretion despite decreased estrogen levels [30]. Sex-hormone-binding globulin levels decrease with decreased estrogen concentration, increasing the free androgen index and reinforcing the imbalance between estrogen and androgens. An increase in bioavailable testosterone can induce fat accumulation in the preadipocytes of visceral fat. Visceral adipocytes express more androgen receptors owing to their downregulation by estrogen. Therefore, visceral fat may become more sensitive to the negative effects of androgens due to a decrease in estrogen levels. In addition, changes in the bioavailability of androgens appear to be predictive of visceral fat accumulation over a 5-year period [31]. Moreover, the incidence of obesity is higher in postmenopausal women, with faster increases in bioavailable testosterone levels than in those with more stable levels; in contrast, women with slower increases in bioavailable testosterone levels experience smaller increases in adipose tissue [31]. Elevated follicle stimulating hormone (FSH) levels may also contribute to this situation because FSH receptors (FSHRs) are expressed in visceral adipocytes. Animal studies have found that FSHR signaling could increase adipocyte lipid synthesis and leptin levels and decrease adiponectin in serum, thus promoting fat accumulation [32].

In the case of surgical menopause, estrogen and progesterone levels suddenly drop. Unlike spontaneous menopause, in which ovarian function slowly declines over several years until menstruation completely stops, an abrupt decline in estrogen levels after surgery may be associated with dysfunction of endothelium, increase in atherosclerotic lipoproteins, and increase in lipid oxidation [33]. In a HUNT-2 study, which is a controlled population-based study, women who had a removed bilateral ovary before the age of 50 years had a higher prevalence of MetS than controls (47% vs. 36% by the International Diabetes Federation (IDF) definition and 35% vs. 25% by the National Cholesterol Education Program-Adult Treatment Panel-III (NCEP ATP III) definition) after adjusting for confounding factors [34]. A population-based cohort study for 12 years found a higher odds ratio for MetS in surgical menopause women—9.7—(95% confidence interval [CI]: 1.8–51.8) than in those with spontaneous menopause. Based on these results, the authors suggested that metabolic disorders were more prevalent in surgical menopausal women than in spontaneous menopausal women [35]. In addition, fasting blood glucose level and 2-h blood glucose level were significantly higher in surgically menopausal women than in spontaneously menopausal women. Additionally, mean systolic blood pressure was higher in spontaneous postmenopausal women than in postmenopausal women after surgery [36].

### 2.3. Metabolic Disorders in Women with Menopause 

**Figure 1:**
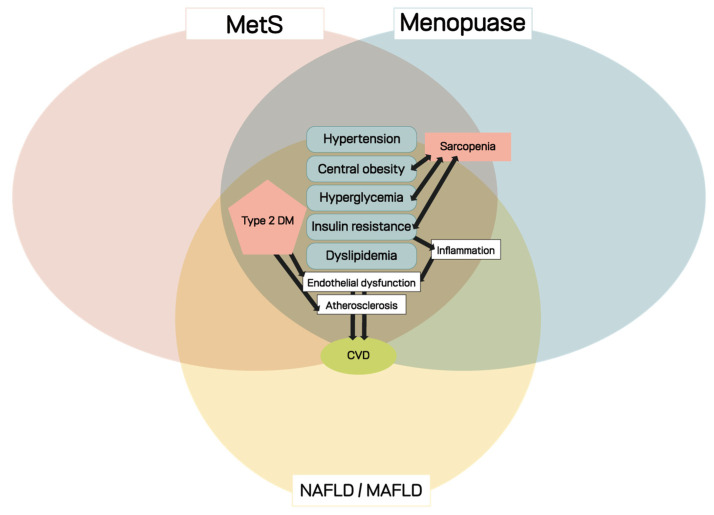
Association of metabolic syndrome, NAFLD/MAFLD, and menopause.

Metabolism is the process of obtaining energy from food made up of proteins, carbohydrates, and fats. The chemicals and enzymes in the digestive system break down and digest food to obtain the energy and nutrients necessary to sustain life. Metabolic disorder refers to a problem in any one of these processes that can occur in various organs and systems. There are various categories of metabolic disorder; especially, during menopause transition, some metabolic disorder could occur because of the change in body composition [37]. Metabolic diseases commonly found in menopause include components of MetS, such as dyslipidemia, impaired glucose tolerance, and type 2 diabetes, which are risk factors for cardiovascular disease [9,37]. This review focuses on MetS and NAFLD/MAFLD, which can chronically affect middle-aged and older adults, especially postmenopausal women (Figure 1).

#### 2.3.1. Definition and Prevalence of MetS

MetS is not a disease but a combination of factors that increase the risk of CVD; it comprises abdominal obesity, dyslipidemia, hyperglycemia, IR, and hypertension [13]. 

In general, the prevalence of MetS is higher in men than in premenopausal women of similar age [38]. However, the trend in MetS prevalence is reversed during menopause, with the prevalence of MetS becoming higher in women than in men [39]. According to the Study of Women’s Health Across the Nation (SWAN), the incidence of MetS increased gradually between 6 years before FMP and 6 years after FMP regardless of age and other known CVD risk factors. Approximately 13.7% of the women in the SWAN cohort had new cases of MetS at the time of FMP [40]. The prevalence of MetS in postmenopausal women reportedly varies by country and race but is estimated to range from 32 to 58%. This is significantly higher than that in premenopausal women [41].

#### 2.3.2. Diagnosis of MetS 

For years, different criteria for MetS were proposed, including those of the World Health Organization (WHO) [42], NCEP-ATP-III [43], American Association of Clinical Endocrinologists (AACE) [44], American Heart Association/National Heart Lung and Blood Institute (AHA/NHLBI) [45], International Diabetes Federation (IDF) [46], and consensus definitions of IDF and AHA/NHLBI [47]. The diagnostic criteria of the WHO and AACE are focused on IR, while those of the NCEP-ATP III, AHA/NHLBI, and IDF include WC as a surrogate marker of central obesity (Table 1). 

In general, MetS is defined based on five criteria: increased WC, hypertension, low high-density lipoprotein-cholesterol (HDL-C), and high triglyceride (TG) concentrations and hyperglycemia. According to the IDF definition, abdominal obesity and two additional criteria are essential for diagnosis of MetS. In 2009, the IDF criteria were updated and became consistent with the recommendations of the AHA/NHLBI [37]. The most notable change was the introduction of three out of the four criteria, except abdominal obesity at the time of MetS diagnosis.

#### 2.3.3. Pathophysiology of MetS

The pathophysiology of MetS is multifactorial and remains unclear. Five diagnostic criteria for MetS have been suggested as definitions but are not limited thereto. Therefore, the IDF consensus group suggested additional parameters associated with MetS that should be included in the research. These additional criteria include abnormal body fat distribution, atherosclerotic dyslipidemia, dysglycemia, IR, vascular dysregulation, pro-inflammatory conditions, prothrombotic conditions, and hormonal factors, which were called “platinum standards”.

Abdominal obesity and IR appear to be the most critical factors in the pathophysiology of MetS and its individual components [48]. Central obesity could be considered an early stage of additional disorders related to MetS. Visceral adipose tissue releases adipocytokines, such as leptin and resistin, inflammatory cytokines, such as interleukin-6 (IL-6) and tumor necrosis factor alpha (TNF-α), thrombogenic factors, such as plasminogen activator inhibitor 1 (PAI-1), and vasoconstrictors, such as angiotensin II. Adipose tissue also contains some protective cytokines, such as adiponectin, interleukins (IL-10, IL-4, IL-13), IL-1 receptor antagonist (IL-1Ra), and apelin. Moreover, the aforementioned substances have anti-inflammatory or insulin-sensitizing effects [49]. 

One of the important roles in the pathophysiology of abdominal adiposity and IR is hyperactivity of the hypothalamic–pituitary–adrenal axis (HPA). Glucose intolerance and IR are known to accompany up to 80% of Cushing’s syndrome patients, but, apart from that, hyper cortisol could result from chronic stress. In peripheral target tissue, IR is induced directly by hypercortisolism in proportion to glucocorticoid levels. These changes can lead to hyperinsulinemia and increased visceral obesity, which can cause dyslipidemia, hypertension, T2DM, and, finally, MetS. Even after multiple adjustments for BMI and age, serum cortisol concentrations in women with MetS were more correlated with leptin levels, as well as components of MetS, such as WC, waist-to-hip ratio (WHR), TG, and total cholesterol, compared to men with MetS. Leptin has been estimated to have a greater effect on the HPA axis in women than in men [50].

Since menopause is an aging phenomenon, there has been debate about whether metabolic disorders are the result of hormonal changes due to menopause or simply a result of aging. A result from an analysis of 1002 women who underwent health checkups every year revealed that the risk of MetS had been increased in menopausal women, even after adjusting for some factors such as BMI and age [51]. All MetS components are significantly associated with postmenopausal status, but only abdominal obesity has a significant association with menopause after adjusting for age [52]. A Swedish study involving 300 postmenopausal women also reported that menopause did not increase the risk of MetS. Therefore, the results so far suggest that both menopause and temporal aging contribute to the elevated risk of metabolic disorders in women after menopause [53].

#### 2.3.4. NAFLD/MAFLD in Menopausal Women

Fatty liver disease (FLD), one of the metabolic disorders, is widespread worldwide and has an estimated prevalence of 25% [54]. In the past, FLD was classified into alcoholic liver disease (ALD) and non-alcoholic fatty liver disease according to alcohol intake. Recently, however, a new term, metabolic associated fatty liver disease, MAFLD has been proposed in 2020 to eliminate the negative effects of alcohol in the name and to emphasize the importance of metabolic abnormalities in fatty liver disease [54,55,56].

NAFLD is diagnosed by the presence of hepatic steatosis confirmed by blood, imaging, or histological examination, without excessive alcohol intake (20 g/day for women and 30 g/day for men) and the absence of other secondary causes of hepatic steatosis [57]. MAFLD could diagnosed when radiological evidence of hepatic steatosis is accompanied by three metabolic abnormalities: overweight/obesity, DM, or metabolic dysregulation [54,55,58]. Metabolic dysregulation is presented in Table 2.

To date, NAFLD and MAFLD have been used interchangeably, although there is some thought that the definition of MAFLD is more effective than for differentiating FLD patients with high risk of progression [54]. NAFLD/MAFLD patients are gradually increasing, and it is reported that about 30% of the general population, 70% of type 2 DM patients, and most obese patients are accompanied by NAFLD/MAFLD [59]. Visceral fat accumulation is one of the characteristics of FLD. Overweight, obesity, dyslipidemia, insulin resistance, type 2 DM, and sedentary behavior accompanied by lack of physical activity, which are closely related to factors of MetS, are the major causes of the increasing trend of NAFLD/MAFLD worldwide [60]. Therefore, in postmenopausal women, the rate of progression of NAFLD/MAFLD and the severity of the disease increase. This is when obesity increases due to changes in lipid transport and lipid metabolism, and the distribution of fat from the subcutaneous to visceral adipose tissue changes, which increases the severity of NAFLD/MAFLD in postmenopausal women. Additionally, when estradiol-mediated gene expression of enzymes involved in lipid metabolism increases NAFLD/MAFLD susceptibility in postmenopausal women, they become more susceptible to NAFLD/MAFLD [59,60].

In postmenopausal women, higher visceral fat is accumulated due to changes in metabolism and body composition, and adipose tissue is more lipolytic after estrogen reduction [23,60]. In addition, expression of housekeeping proteins related with the management of hepatic lipid is altered, contributing to the pathogenesis of NAFLD. Excessive hepatic steatosis accelerates NAFLD progression by causing hepatic insulin resistance, oxidative stress, and inflammation [60]. 

#### 2.3.5. Other Considerations

One opinion that has recently received attention is that sarcopenia and MetS are related. These two conditions should also be considered more closely as they are more prevalent in postmenopausal women. Skeletal muscle is relevant to major organs involved in insulin-induced glucose metabolism, and loss of muscle mass is associated with IR and MetS [61,62]. Sarcopenia is defined as a decrease in the quantity and quality of skeletal muscle and muscle function and is usually common in the elderly (Table 3) [62]. Skeletal muscle loss and intramuscular fat accumulation cause abnormal muscle contractility and metabolic abnormalities and are thought to be related to various factors, such as oxidative stress, inflammatory cytokines, mitochondrial dysfunction, and IR [63].

A positive correlation is emerging between sarcopenia and obesity. When lean body mass decreases, physical activity decreases and risk of obesity increases. The synergistic effect of fat tissue and muscle led to introduction of a new and important concept called sarcopenic obesity [71]. Sarcopenic obesity is defined as a condition accompanied by a decrease in skeletal muscle mass, muscle weakness, and an increase in visceral adipose tissue. International standard diagnostic criteria have not yet been established [72].

## 3. Diagnosis of Metabolic Disorders in Menopausal Women

### 3.1. History Taking and Physical Examination

The clinical diagnosis of menopause is the absence of menstruation for 12 months, and, since FMP, irregular menstrual cycle and recent weight change can be clues for diagnosing menopause and MetS after menopause. History taking is important. Weight gain is the main complaint in middle-aged women, especially perimenopausal women [73]. The prevalence of obesity is reportedly higher in postmenopausal women than in premenopausal women. The prevalence of central obesity was 65.5% among women aged 40–59 years and 73.8% among those aged ≥60 years in the United States in 2008 [74]. Absolute weight gain in perimenopausal women appears to be fundamentally related to aging rather than menopause itself [73]. However, redistribution of body fat, accumulation of visceral fat, increase in WC, and changes in body shape appear to be dependent on menopause [75,76]. Visceral adipose tissue poses a greater health risk than subcutaneous fat and is usually an independent cause of CVD. Therefore, abdominal obesity increases IR, and, consequently, risk of diabetes and MetS [77].

Information that could be found through physical examination includes BMI, WC, and WHR. BMI and WC differ depending on the organization, but there are differences in the standards according to sex and race.

Blood pressure is a diagnostic criterion for MetS. Although there is a slight difference in blood pressure values, the diagnostic criteria are considered to be met if 130–140 mmHg or more for systolic, 85–90 mmHg or more for diastolic, or if the patients are taking antihypertensive drugs [42].

### 3.2. Laboratory Examination

Among the criteria for MetS, fasting glucose, HDL-C, and TG levels can be measured by serum blood tests. The standards for each institution are generally consistent. 

In addition, apolipoprotein B, non-HDL-C, low-density lipoprotein-cholesterol, free fatty acid, homeostatic model assessment of insulin resistance (HOMA-IR), C-reactive protein, inflammatory cytokines, fibrinolytic factors, clotting factors, and microalbuminuria can be measured to identify various indicators corresponding to the aforementioned “platinum standards”; however, they are not essential for diagnosis [47].

### 3.3. Imaging Test

Imaging tests are not essential for diagnosing MetS. Physicians can utilize dual-energy X-ray absorptiometry to check the distribution of body fat, computed tomography, or magnetic resonance imaging to determine central fat distribution, and magnetic resonance spectroscopy to check the liver fat content [47]. 

### 3.4. Other Biomarkers Associated with MetS

BMI, WC, and WHR increase in men and women with age, and the trend changes between men and women in their fifties (50–59 years) [78]. Perimenopausal women might undergo a more abrupt change in lean and fat mass than men [79]. In perimenopausal women, waist to height ratio (WHtR), WHR, body roundness index (BRI), and lipid accumulation product (LAP) could predict CVD and its risk factors [80,81,82,83]. The method for calculating each value is as follows [28]

- BMI: Weight (kg)/square of height (m)
- WHR: WC (cm)/hip (cm);
- WHtR: WC (cm)/height (cm);
- LAP for women: WC (cm) − 58 × TG (mmol/L) [84];
- BRI: 364.2 − 365.5 × $\sqrt{1-\left(\frac{{\left(wc/\left(2\pi \right)\right)}^{2}}{{\left(0.5\;height\right)}^{2}}\right)}$ [85];
- Body shape index: [WC (cm)]/[BMI 2/3 × height (cm)1/2] [86].

Farahmand et al. recommended that clinicians use some easily measurable obesity indices, including WC, WHtR, WHR, in their routine practice. Measuring the obesity index and monitoring changes in the obesity index throughout an individual’s lifespan may help to predict the risk early and allow for intervention at an appropriate time to prevent morbidity and mortality associated with these changes in postmenopausal women [28].

## 4. Management of Metabolic Disorders in Menopausal Women

Among the components of MetS, drug treatment should be administered according to the medical diagnostic criteria for hypertension, dyslipidemia, and glucose metabolism abnormalities, including T2DM.

Hormone replacement therapy (HRT), provided the selected progestin does not antagonize estrogen action, may improve fat mass and distribution, dyslipidemia, and insulin sensitivity in postmenopausal women [38]. A meta-analysis performed by Salpeter et al. demonstrated that HRT for MetS has a beneficial effect [87]. In 107 trials that enrolled 33,315 subjects, HRT increased lean body mass and reduced abdominal fat, HOMA-IR, and onset of new diabetes in previously nondiabetic postmenopausal women. Additionally, HRT decreased fasting blood glucose levels (11.5%, 95% CI 5.1–18.0%) and HOMA-IR (35.8%, 95% CI 19.8–51.7%, *p* = 0.007) in women with diabetes with menopause compared to controls. It also improved metabolic functions, including increasing HDL-C (5.1%, 95% CI 3.6–6.7%), while decreasing LDL-C (−11.0%, 95% CI, −12.3 to −9.6%), LDL-C/HDL-C ratio (−15.7%, 95% CI −18.0 to −13.5%), Lp(a) (−25%, 95% CI −32.9 to −17.1%), fibrinogen (−5.5%, 95% CI −7.8% to −3.2%), and PAI-1 (−25.1%, 95% CI −33.6% to −15.5%) in postmenopausal women with and without diabetes [87]. Generally, oral HRT was more advantageous than percutaneous HRT. In a subgroup analysis, oral HRT resulted in significant decreases in PAI-1 (−27.0%, 95% CI: −38.0 to −22.0%), whereas transdermal HRT had no decreasing effect on PAI-1 (−3.0, 95% CI −23.0 to 35.0%, *p* = 0.03) [87]. The effect of HRT on blood pressure was negligible. The authors concluded that, although oral HRT had a significant beneficial effect on most components of MetS, it could adversely affect c-reactive protein (CRP) and TG concentrations, possibly weakening the beneficial effects of HRT on the cardiovascular system [87]. A more recent review paper also suggested that menopausal hormones have beneficial properties for the components of MetS, but it cautioned that menopausal hormone therapy (MHT) should be used carefully in patients with MetS and that a thorough evaluation be conducted before initiation of MHT [87].

In addition, the effect of MHT on cardiovascular diseases remains controversial. According to the results of most systematic and meta-analyses, HRT does not affect incidence of CVD or even increase the risk of cardiovascular events (Table 3) [88,89,90,91,92,93,94,95]. As a result, patient characteristics and initiation timing are important factors for the preventive effect of HRT, and it is not recommended that MHT be used for prevention of CVD (Table 4).

In conclusion, the effect of MTH on CVD is associated with the baseline characteristics of menopausal women, such as age, BMI, and presence of MetS, and HRT types, such as oral and transdermal. It is necessary to provide further evidence through better-designed studies.

## 5. Prevention of Metabolic Disorders in Menopausal Women

Strategies for preventing metabolic diseases in postmenopausal women include lifestyle modifications, such as physical activity and calorie-controlled diet, pharmacotherapy, bariatric surgery, traditional health practices, and medicines. 

Diet is a key factor in management of MetS [96]. A high-calorie diet rich in carbohydrates and saturated fats is known to cause obesity and increase abdominal obesity. Because energy-dense foods are more accessible, easier to prepare, and less expensive than fresh-product-rich diets, socioeconomic factors might play an essential role in modern obesity [97].

Several studies have established the relationship between dietary patterns and MetS risk in postmenopausal women. The Framingham Nutrition Study found that women who consumed more total fat, saturated fat, monounsaturated fats, and alcohol and less fiber and micronutrients, such as calcium, selenium, vitamin C, vitamin B-6, folate, vitamin E, and β-carotene excluding vitamin B-12 had a higher risk (2–3 times) of abdominal obesity and overall MetS during a 12-year follow-up period (odds ratio: 2.3 (95% CI: 1.2–4.3) and 3.0 (95% CI: 1.2–7.6)) [96]. In a cross-sectional study of 4984 Korean women aged 30–79 years, traditional healthy diets, such as more intake of seafood, seaweeds, grains, dairy products, fresh vegetables, and fruits, and less consumption of fast foods, animal fats, sweets, and fried foods were associated with a reduced risk of MetS. The prevalence of MetS in postmenopausal women was significantly lower in women who ate a healthy diet than in those who ate a Western diet (OR = 0.60; 95% CI: 0.41–0.86; *p* = 0.004) [98]. 

An energy-restricted diet combined with physical activity uses the energy stored in adipose tissue and results in gradual decreases in body weight. In the Women’s Health Initiative study, there was a 17% reduction in the risk of MetS at 3 years (OR = 0.83; 95% CI: 0.59–1.18) but not at 1 or 6 years. Additionally, cholesterol reduction combined with antihypertensive therapy was 19% lower in the low-fat diet group than in the control group at 1 year (OR = 0.81; 95% CI: 0.60–1.09; *p* = 0.016) [99]. Another study by Rodriguez-Cano et al. confirmed that a calorie-restricted diet resulted in a positive effect on MetS components in postmenopausal women. Absence of high-energy refined grains was associated with increased probability of normal fasting blood glucose. And low-fat diet was related to reduced diastolic blood pressure and increased HDL-C levels [100]. These results suggest that diet is important in prevention of MetS in postmenopausal women [60]. 

In addition, nutrition and supplementation, such as foods rich in vitamin D, omega-3 fatty acids, antioxidants, phytochemicals, and probiotics, are recommended for MetS, but further research is needed [4].

## 6. Conclusions

In this review paper, the pathophysiology, diagnosis, treatment, and prevention of metabolic diseases that can occur during menopause, especially MetS and NAFLD/MAFLD, were reviewed. The components of MetS appear to be independent; however, central obesity and insulin resistance are at the center of the pathology. The importance of MetS and NAFLD/MAFLD is further emphasized because they increase the risk of cardiovascular events and cause long-term health problems in middle-aged women who live 30–40 years after menopause. In the case of diseases such as hypertension and diabetes, drug treatment is implemented, but lifestyle modifications, such as a calorie-restricted diet and increased physical activity, are also important for management and prevention of MetS and NAFLD/MAFLD. Recently, new facts regarding the relationship between diseases of skeletal muscle metabolism, such as sarcopenia and MetS, have been revealed. Further research is required on this topic. It is hoped that strategies will be established to detect and prevent MetS early and accurately before and after menopause.

## Figures and Tables

**Table 1:** Diagnosis of MetS [7].

	World Health Organization (1998) [32]	NCEPT: ATPIII (2001) ^a^ [33]	AACE (2003) ^b^ [34]	AHA/NHLBI (2004) ^c^ [35]	IDF (2005) ^d^ [36]	Consensus Definition IDF and AHA/NHLBI (2009) [37]
Required	IR T2DM IFG Fasting glucose ≥ 110 mg/dL 2-h glucose ≥ 140 mg/dL		Impaired glucose tolerance (IGT) or IFG		Elevated WC (depending on population ^f^) in European women ≥ 80 cm ^g^	
Number of abnormalities	And ≥2 of:	≥3 of:	Any of the following based on clinical judgment:	≥3 of:	And ≥2 of:	≥3 of:
Glucose		Fasting glucose ≥ 110 mg/dL (includes diabetes)	- IGT or IFG (but not diabetes) - Other features of IR (includeing family history of DM, polycystic ovary syndrome, sedentary lifestyle, advancing age, and ethnic groups susceptible to T2DM.)	Fasting glucose ≥ 100 mg/dL or drug treatment of elevated glucose	Fasting glucose ≥ 100 mg/dL or previously diagnosed T2DM	Fasting glucose ≥ 100 mg/dL or drug treatment of elevated glucose
HDL cholesterol	HDL-C < 50 mg/dL	HDL-C < 50 mg/dL	HDL-C < 50 mg/dL	HDL-C < 50 mg/dL or specific treatment for this lipid abnormality ^e^	HDL-C < 50 mg/dL or specific treatment for this lipid abnormality ^e^	HDL-C < 50 mg/dL or specific treatment for this lipid abnormality ^e^
Triglycerides	≥150 mg/dL	≥150 mg/dL	≥150 mg/dL	≥150 mg/dL or specific treatment for this lipid abnormality	≥150 mg/dL or specific treatment for this lipid abnormality	≥150 mg/dL or specific treatment for this lipid abnormality
Obesity	Central obesity: WHR > 0.85 or BMI > 30 kg/m^2^	WC > 88 cm	BMI ≥ 25 kg/m^2^	WC > 88 cm		WC ≥ 80 cm (in European women) (depending on population ^f^)
Hypertension	≥140/90 mmHg	≥130/85 mmHg	≥130/85 mmHg	≥130/85 mmHg or antihypertensive drug treatment	≥130/85 mmHg or antihypertensive drug treatment	≥130/85 mmHg or antihypertensive drug treatment
Other	Microalbuminuria: urinary albumin excretion ratio ≥ 20 μg/min or albumin: creatinine ratio ≥ 30 mg/g					

^a^ National Cholesterol Education Program Adult Treatment Panel III. ^b^ American Association of Clinical Endocrinologists. ^c^ American Heart Association/National Heart Lung and Blood Institute. ^d^ International Diabetes Federation. ^e^ Fibrates and nicotinic acid are the drugs most used to elevate TG and reduce HDL-C levels. Patients taking one of these drugs were presumed to have high TG and low HDL levels. ^f^ 80 cm or greater (Asian, Japanese, Chinese, Middle East, Mediterranean, Sub-Saharan Africa, Ethnic Central, and South America). ^g^ If BMI is >30 kg/m^2^, central obesity can be assumed and WC does not need to be measured. T2DM, type 2 diabetes mellitus; IFG, impaired fasting glucose; IGT, impaired glucose tolerance; TG, triglycerides; HDL-C, high-density lipoprotein cholesterol; BMI, body mass index, WC, waist circumference. Adapted with permission from Ref. [7]. Copyright 2015, copyright Elsevier Books.

**Table 2:** Metabolic dysregulation in diagnosis of MAFLD (two or more) [54].

Waist circumference	≥102 cm (men), 88 cm (women)
Blood pressure	≥130/85 mmHg or specific drug treatment
TG	≥1.70 mmol/L or specific drug treatment
HDL-C	<1.0 mmol/L (male), <1.3 mmol/L (female)
Prediabetes	fasting glucose levels: 5.6–6.9 mmol/L 2-h post-load glucose level 7.8–11.0 mmol/L HbA1c 5.7% to 6.4%
Homeostasis model assessment-insulin resistance (HOMA-IR) score	≥2.5
C-reactive protein (CRP)	2 mg/L

**Table 3:** Diagnostic criteria for sarcopenia [64].

	EWGSOP	ESPEN SIG	IWGS	Sarcopenia with Limited Mobility	FNIH	AWGS
Muscle mass (DXA)	SMI M: ≤7.26 kg/m^2^ F: ≤5.54 kg/m^2^	LM total kg	SMI M: ≤7.23 kg/m^2^ F: ≤5.67 kg/m^2^	SMI M: ≤6.81 kg/m^2^ F: ≤5.18 kg/m^2^	LM_App_ M: <19.75 kg F: <15.02 kg LM_App_/BMI M: <0.789 F: <0.512	ASM DEXA M: <7.0 kg/m^2^ F: <5.4 kg/m^2^
Muscular function Handgrip	Kg BMI	-	-	-	M: <26 kg F: <16 kg kg/BMI M: <1 F: <0.56	M: <28 kg F: <18 kg
Waking speed	<0.8 m/s	<0.8 m/s	<1 m/s	<1 m/s 6MWT < 400 m	-	<1 m/s 6MWT Or 5-time chair stand test: ≥12 s or Short physical performance battery: ≤9
Timed up-and-go test	>10 s	-	-	-	-	

EWGSOP, European Working Group on Sarcopenia in Older People [65]; ESPEN SIG, Special Interest Group “cachexia-anorexia in chronic wasting diseases” [66]; IWGS, International Working Group on Sarcopenia [67]; Sarcopenia with limited mobility, Society for Sarcopenia Cachexia and Wasting Disorders [68]; FNIH Sarcopenia Project [69]; and AWGS [70]. LM, lean mass; App, appendicular; SMI, skeletal muscle mass index; BMI, body mass index; 6 MWT, 6-min walking test; M, male; F, female.

**Table 4:** Effects of MHT on cardiovascular systems according to the results of several meta-analyses.

Meta-Analysis (Year)	No. of Trials (No. of Participants)	Conclusions
Kim, et al. (2020) [92]	RCT (26) Observational (47)	RCTs and observational studies both showed that MHT was associated with an increased risk of VTE and PE, although only the RCTs revealed an increased risk of stroke among those administered MHT. A decrease in the risk of MI due to MHT was identified in the observational studies, but the RCTs did not show this association. The risks and benefits of MHT may vary depending on the characteristics of the women who are treated. MHT is not recommended for prevention of chronic disease. However, it may be beneficial in CVD and for mortality in postmenopausal women with severe menopause symptoms after sufficient consideration of underlying diseases and timing of treatment initiation. It may also suggest use of non-oral MHT in women at high risk of VTE and stroke.
Oliver-Williams et al. (2019) [95]	33 (2,588,327)	Use of low-dose oral and transdermal hormone therapy seems to be safe with respect to CVD risk in women in menopausal transition and within the first years (e.g., 10 years) after menopause onset. In women with increased baseline thromboembolic risk, alternative non-hormonal medications are suggested as first-line treatment, and transdermal estradiol alone or with micronized progesterone only should be considered when the previously mentioned options are not effective. When MHT is initiated >10 years since menopause onset (>60 years old), because of greater absolute risks of coronary heart disease, stroke, and venous thromboembolism, it should be used for the shortest possible time and in the lowest possible dose and should be administered transdermally. However, an individualized treatment approach including baseline CVD risk assessment should be applied when prescribing MHT.
Nudy et al. (2019) [94]	31 (40,521)	When a study with a starting time point of <60 years of age was defined as a younger initiation trial and a study with an average age of >60 was defined as an older initiation trial, younger initiation of MHT may be effective in reducing mortality and cardiac events. However, those in whom younger initiation of HRT was conducted remained at an increased risk of stroke, TIA, and systemic embolism, and this risk increased as average age increased. Younger menopausal women using MHT for treating vasomotor symptoms do not appear to be at an increased risk of mortality or cardiovascular events.
Boardman et al. (2015) [93]	19 (40,410)	There is strong evidence that treatment with hormone therapy in postmenopausal women overall, for either primary or secondary prevention of cardiovascular disease events, has little if any benefit and causes an increase in the risk of stroke and venous thromboembolic events. MHT in both primary and secondary prevention conferred no protective effects for all-cause mortality, cardiovascular death, non-fatal myocardial infarction, angina, or revascularization.
Yang et al. (2013) [88]	10 (38,908)	There is no effect on CVD, such as myocardial infarction, coronary events, and even cardiac and total death, after combination therapy of estrogen combined with medroxyprogesterone acetate therapy. Estrogen monotherapy was related to a 27% increased risk for incident stroke. MHT should not be recommended in women with postmenopause for the purpose of preventing cardiovascular disease.
Sare et al. (2008) [89]	31 (44,113)	MHT is related to an increased risk of CVD (stroke) and VTE. Adding progesterone to estrogen increases the risk of VTE 2-fold. There is no effect of MHT on coronary heart diseases, such as myocardial infarction, unstable angina, and sudden death from cardiac causes. MHT could not be recommended for long-term prophylaxis of vascular events in most women.
Canonico et al. (2008) [90]	9 (38,779)	Oral estrogen increases risk of VTE, especially in obese women and during the first year of treatment. Transdermal estrogen might be safe in VTE.
Magliano et al. (2005) [91]	7 (32,523)	There is no effect of MTH on nonfatal acute myocardial infarction, coronary heart disease mortality, or all-cause mortality. MTH is increasing the risk of stroke in women with menopause. Hormone therapy for reduction or prevention of CVD risk is not supported.

RCT: randomized controlled trial, MHT: menopausal hormone therapy, VTE: venous thromboembolism, PE: pulmonary embolism, CVD: cardiovascular disease, TIA: transient ischemic attack.
